# Perception of temporal structure in speech is influenced by body movement and individual beat perception ability

**DOI:** 10.3758/s13414-024-02893-8

**Published:** 2024-05-20

**Authors:** Tamara Rathcke, Eline Smit, Yue Zheng, Massimiliano Canzi

**Affiliations:** 1 Department of Linguistics, University of Konstanz, Konstanz, 78464 Baden-Württemberg Germany; 2 The MARCS Institute for Brain, Behaviour and Development, Western Sydney University, Street, Penrith, 2751 NSW Australia; 3 Department of Psychology, University of York, York, YO10 5DD UK; 4 Department of Hearing Sciences, University of Nottingham, Nottingham, NG7 2RD UK

**Keywords:** Perceptual regularization, Beat perception ability, Sensorimotor synchronization

## Abstract

The subjective experience of time flow in speech deviates from the sound acoustics in substantial ways. The present study focuses on the perceptual tendency to regularize time intervals found in speech but not in other types of sounds with a similar temporal structure. We investigate to what extent individual beat perception ability is responsible for perceptual regularization and if the effect can be eliminated through the involvement of body movement during listening. Participants performed a musical beat perception task and compared spoken sentences to their drumbeat-based versions either after passive listening or after listening and moving along with the beat of the sentences. The results show that the interval regularization prevails in listeners with a low beat perception ability performing a passive listening task and is eliminated in an active listening task involving body movement. Body movement also helped to promote a veridical percept of temporal structure in speech at the group level. We suggest that body movement engages an internal timekeeping mechanism, promoting the fidelity of auditory encoding even in sounds of high temporal complexity and irregularity such as natural speech.

## Introduction

The perception of time, timing, and temporal structure in speech is considered foundational for language mastery (Goswami, 2011; Pagliarini et al., 2020). Yet evidence pertaining to the corresponding perceptual ability to encode the temporal occurrence and the duration of speech units is scarce and somewhat conflicting (see White and Malisz (2020) for an overview). This empirical issue stands in stark contrast to detailed accounts of timing in speech production that is guided by precise time constraints (Browman and Goldstein, 1992; Byrd and Krivokapić, 2021; Pouplier, 2020). The present report addresses the issue of temporal perception in speech by studying the influence of the ability to track the temporal structure in rhythmic sounds like music, which is known to be individually variable (Dalla Bella et al., 2017; Fiveash et al., 2022; Harrison & Müllensiefen, 2018a), and the influence of a concurrent body movement that is known to support the perceptual encoding of the temporal structure in complex sounds (Chemin et al., 2014; Su and Pöppel, 2012).

The fact that the subjective experience of time flow in speech deviates from the sound acoustics in rather substantial and complex ways was discovered in the early days of speech perception research (Darwin & Donovan, 1980; Donovan & Darwin, 1979; Lehiste, 1973, 1977; Morton et al., 1976; Scott et al., 1985). This discovery owes to the pervasive idea that temporal isochrony - or equal spacing of time intervals - is the governing principle of spoken language and the source of its rhythmicity (Abercrombie, 1967; Classe, 1939; Ladefoged & Johnson, 1975). As soon as the tools of acoustic speech analyses became widely available, the isochrony idea was identified as inadequate, and the focus of research shifted toward the systematic nature of the discrepancy between the perception and the acoustics of time series in speech (Dauer, 1983; Roach, 1982). A range of methods was employed to study the systematic gap between the perceptual experience and the signal acoustics, giving rise to two key findings. First, the time point of the onset of a perceived speech event tends to lag behind the acoustic onset of the corresponding speech unit (Cooper et al., 1986; Fowler et al., 1988; de Jong, 1994; Fox & Lehiste, 1987; Marcus, 1981; Morton et al., 1976; Patel et al., 1999). This peculiarity of speech perception is commonly referred to as the perceptual center effect (or the p-center (Morton et al., 1976)). The p-center of a speech unit such as a syllable or a word is defined as its “psychological moment of occurrence” (Morton et al., 1976 p.405). Its timepoint does not consistently coincide with any specific acoustic markers of the speech signal (Marcus, 1981; Scott, 1998; de Jong, 1994) and can be influenced by several aspects of the speech unit in question, including its structural, temporal and acoustic properties (Pompino-Marschall, 1989; Scott, 1998; Ryan, 2014; Harsin, 1997; Howell, 1988).

Second, the perception of temporal intervals in speech is prone to regularization. When creating patterns of clicks or producing finger taps to represent the subjectively experienced time intervals of speech, listeners produce more regular patterns than the ones measured acoustically (Darwin & Donovan, 1980; Donovan & Darwin, 1979; Benadon, 2014; Scott et al., 1985; Rathcke et al., 2021). Long intervals in speech tend to be perceived shorter than their acoustic duration while short intervals are perceived longer than they physically are Lehiste (1973). The magnitude of these effects can scale up to 150 ms or 30% of the corresponding interval duration (Donovan & Darwin, 1979; Lehiste, 1973, 1977), which is six times higher than typically observed just-noticeable differences in sound durations (Friberg and Sundberg, 1995; Quené, 2007). Intriguingly, regularization is limited to the perceptual judgements of time intervals in speech and does not seem to apply to similarly timed non-speech sounds that are evaluated in a more veridical fashion (Darwin and Donovan, 1980; Lehiste, 1973; Scott et al., 1985; Benadon, 2014).

There are currently no satisfactory explanations of the two perceptual findings. The concept of the p-center has “no explanatory power” of its own (Morton et al., 1976 p.408). Rather, it is assumed to capture the perceptual experience of spoken language (Morton et al., 1976; Scott, 1998). After more than 40 years of research, the exact location of the p-center in spoken language has remained elusive (Villing et al., 2011). Some researchers follow the suggestion (Allen, 1972) that the onset of a speech unit (commonly referred to as its “beat” (Allen, 1972; Rapp-Holmgren, 1971)) may be like a “broad slur” rather than a single point in time (Allen, 1972; Benadon, 2014), with some onsets having a higher resolution of the subjective time of occurrence than others Villing et al. (2011). Similarly, perceptual regularization of time intervals in speech is far from being comprehensively described or well understood. Early accounts of the effect sought its origin in the realm of just-noticeable differences in duration of speech units, arguing “if you cannot tell them apart, they must be alike” (Lehiste, 1977 p.257). However, later work showed that the perceptual system is highly sensitive to the even slightest changes in sound duration - but only if sounds are simple, i.e., consisting of one consonant or one vowel. Just-noticeable differences (JNDs) of such simple speech sounds can range from minimally 6 ms (for shorter sounds) up to about 5% of longer sound duration (Friberg and Sundberg, 1995; Quené, 2007). These thresholds are considerably lower than the differences between successive speech intervals that showed perceptual regularization in previous work (Darwin & Donovan, 1980; Donovan & Darwin, 1979; Benadon, 2014; Scott et al., 1985). Regularization has further been discussed as a perceptual illusion of isochrony that does not reflect the veridical physical stimulus but rather “the underlying object, in this case an underlying regular beat” (Darwin and Donovan, 1980 p. 78). The phenomenon has also been argued to arise as a response bias due to an increasingly difficult task (Scott et al., 1985; Benadon, 2014), though it is unclear why judging speech intervals would be more difficult than judging non-speech intervals of the exact same temporal structure. A process of perceptual compensation for a common speech production tendency toward unit-final lengthening has also been named as a possible source of regularization (Benguerel & D’Arcy, 1986), given the observation that particularly decelerating speech intervals are regularized (Benguerel & D’Arcy, 1986; Lehiste, 1973). While this might be a viable explanation for the regularization processes observed in metrically regular speech (Benguerel & D’Arcy, 1986; Lehiste, 1973), the account appears too simplistic in the context of the well-attested irregularity of natural speech that goes beyond incrementally increasing duration of successive intervals (Jadoul et al., 2016).

Moreover, little research has previously addressed the role of individual listener traits in perceptual regularization. Existing work either misses to capture individual perceptual abilities or does not identify and measure those skills relevant to the task at hand (Darwin & Donovan, 1980; Donovan & Darwin, 1979; de Jong, 1992; Cooper et al., 1986; Lehiste, 1973, 1977; Marcus, 1981; Morton et al., 1976; Pompino-Marschall, 1989; Scott et al., 1985). A previous study suggested that listeners with a high rhythmic skill in music may be less prone to regularization (Benadon, 2014), though the study was not designed to assess listeners’ rhythmic skill. Rather, individual abilities were determined post-hoc from participants’ performance with piano stimuli that were matched in pitch and timing to the speech stimuli of the study. Rhythmic skill entails production, perception as well as memory-based processing of timing patterns involving the beat and high-level rhythmic structures (Fiveash et al., 2022). All aspects of this skill are known to be highly variable across individuals (Fiveash et al., 2022; Harrison & Müllensiefen, 2018a) and might be involved in speech and language processing (Schön & Tillmann, 2015). We hypothesize that among many aspects of rhythmic abilities that may give rise to music sophistication (Dalla Bella et al., 2017; Fiveash et al., 2022), beat perception, in particular, would transfer to the perception of temporal structure in spoken language (Lagrois et al., 2019). Beat perception involves the ability to track timing regularities of sounds with a complex temporal structure such as music (Harrison & Müllensiefen, 2018a; Dalla Bella et al., 2017; Fiveash et al., 2022). Cross-cultural research has found that tracking the beat is a crucial component of the human musical experience (Anglada-Tort et al., 2022; Savage et al., 2015; Jacoby et al., 2021), with an open question if the ability is present in other species (Bouwer et al., 2021; Honing et al., 2018; Patel & Iversen, 2014; Ravignani et al., 2019). Genetic research has linked the ability to entrain to rhythms to specific genotypes (Niarchou et al., 2022). Thus, beat perception might be a universal cognitive ability with arguably deep evolutionary roots (Darwin, 1871; Patel & Iversen, 2014) which is important for temporal processing of auditory events (Bouwer et al., 2016; Patel & Iversen, 2014; Rankin et al., 2009) as well as for social bonding by synchronizing movements together to a beat (Honing et al., 2015). A beat impairment has been suggested to arise from an impaired internal timekeeping mechanism (Tranchant & Peretz, 2020) that we expected to show similar effects across language and music domains (Lagrois et al., 2019). It is not yet established if, and to what extent, an individual’s beat perception ability impacts regularization of temporal intervals in speech.

A growing body of research indicates that synchronized movement affects the perception of temporal structure in a range of sounds (Chemin et al., 2014; Manning & Schutz, 2013; Phillips-Silver & Trainor, 2005, 2007; Su & Pöppel, 2012). Body movement while listening has been shown to enhance sound encoding at the neural level (Nozaradan et al., 2016), with neural motor networks routinely activating during beat perception (Grahn & Brett, 2007; Grahn & Rowe, 2013; Zatorre et al., 2007), even when no movement is involved in the perception task itself (Merchant et al., 2015). These findings suggest a strong link between auditory perception and motor action, though its role in the perception and processing of spoken language has rarely been addressed (Falk & Dalla Bella, 2016). Our previous work has shown that regularization occurs only if temporal intervals between speech units are evaluated after listening, i.e., asynchronously (Rathcke et al., 2021). It is absent if participants are asked to keep in time with a concurrent speech signal (e.g., by tapping along with the beat of spoken sentences). In this case, perceptual tracking of temporal intervals between the onsets of speech units (i.e., the beats) is veridical and mapped quite precisely onto the duration of intervocalic intervals. Without this auditory-motor concurrency, participants’ temporal estimation drifts away from the duration of intervocalic intervals, becomes more regularized and shifts towards individually preferred time-keeping rates (Rathcke et al., 2021). In this regard, beat perception in language shows sensorimotor benefits comparable to those attested for beat perception in other kinds of sound (Chemin et al., 2014; Nozaradan et al., 2016; Su & Pöppel, 2012), though very little is known about potential carry-over benefits of synchronized movement to non-synchronized perception of the temporal structure in speech. It is unclear whether or not the perceptual representation of temporal intervals between speech units would remain veridical after a short synchronization phase had finished. An empirical answer to this question is particularly important for a comprehensive account of the movement effect on perception, given that natural speech lacks isochrony while existing studies document the perceptual benefit primarily with simple temporal structures that are built around isochrony. We can hypothesize that synchronized movement during exposure to complex sounds supports perceptual encoding of their temporal structure (Chemin et al., 2014; Nozaradan et al., 2016; Su and Pöppel, 2012). However, pertinent evidence for spoken language is currently lacking.

Concerning the veridicality of timing in spoken language, previous work indicates that vowel onsets mark the onsets of perceptually prominent events representative of temporal beat structure in speech (Rathcke et al., 2021), cf. Benadon (2014). Notably, the p-center has often been discussed as approximating vowel onsets (Pompino-Marschall, 1989; Scott, 1998; Ryan, 2014; Harsin, 1997; Howell, 1988). Vowels have a unique status in the phonological system of languages. On the one hand, they are acoustically salient and have relatively high energy forming local sonority peaks in the amplitude envelopes of speech signals (Morgan & Fosler-Lussier, 1998; Wang & Narayanan, 2007). On the other hand, they are important phonological elements of language systems defined by their tendency to constitute syllable nuclei and act as the core units of temporal structure in many languages of the world. Even though our previous work has shown that neither local acoustic intensity maxima nor linguistic syllable onsets serve as targets of sensorimotor synchronization in natural speech (Lin & Rathcke, 2020; Rathcke et al., 2021), an open question remains if this is also true for the perception without concurrent movement. Local intensity maxima, vowel and syllable onsets all represent some veridical (acoustic, linguistic) aspects of speech that have not been compared in previous perception studies. We hypothesize that sensorimotor synchronization during listening would support a more veridical encoding of speech timing while reducing or completely eliminating perceptual regularization.

The ability to benefit from synchronized movement during the encoding of the temporal structure might also be individually variable and dependent on the level of rhythmic skill. Many studies have shown that individuals vary in their sensitivity to the beat, which is the key prerequisite for effective synchronization (Grahn & Rowe, 2009; Harrison & Müllensiefen, 2018a; McAuley et al., 2006). For example, Su and Pöppel (2012) asked musically trained and untrained listeners to track the beat period of various auditory sequences that contained omissions of temporally predictable tones and to replicate the perceived beat period either after passive listening or after a period of synchronized exposure. The results demonstrated that synchronized movement assisted beat tracking in listeners without musical training. Musically trained listeners showed comparable performance on both types of tasks, possibly due to an enhanced ability to generate internal representations of the temporal beat structure (Grahn & Rowe, 2009). Previous research indicates that the beat alignment sensitivity forms part of a broader individual phenotype that includes a range of sensorimotor and timekeeping skills (Dalla Bella et al., 2017; Fiveash et al., 2022). Given that synchronized movement relies on a strong beat perception ability, the final question of the present study asks if individuals with different levels of this rhythmic ability would vary with regards to a perceptual benefit from synchronized movement.

The experiment consisted of two tasks that tested temporal perception in linguistic and musical phrases. Participants first performed the linguistic perception task, in which they were presented with repetitions of spoken sentences and asked to either keep quiet during listening (*Listen-Only* exposure) or to move in time with what they perceived to be the beat of the sentence (*Listen-and-Tap* exposure). Both exposure types were then followed by a longer silent pause after which the participants were presented with a drumbeat-based version of the sentence they had previously heard, with the task to make a speeded decision if the temporal structure of the sentence and the time series of drumbeats were same or different. The pause between the last repetition of a sentence and its drummed version was introduced to prevent participants from adopting a synchronization-continuation strategy (Repp et al., 2008; Repp & Keller, 2004; Wing & Kristofferson, 1973) when making perceptual decisions in the synchronized condition. The time series of drumbeat timings included fully isochronous intervals in contrast to veridical representations of different time intervals in the test sentences, comparing linguistic timescales (spanning inter-syllabic or inter-vocalic onsets) and acoustic landmarks (spanning local intensity maxima). The music task used in this experiment was the Computerized Adaptive Beat Alignment Test (CA-BAT) (Harrison & Müllensiefen, 2018a, b). CA-BAT examines individual beat perception ability (BAT ability) by asking listeners to spot temporal mismatches between a metronome beat and a musical extract.

In summary, the present study was designed to investigate the following research questions:

- Does an individual’s beat perception ability impact their perceptual tendency toward temporal regularization in speech? We hypothesized that an enhanced beat perception skill would transfer to language, with a lower tendency toward regularization in rhythmically skilled listeners (Benadon, 2014).
- Does sensorimotor synchronization lead to a robust, veridical encoding of temporal structure in speech? We hypothesized that sensorimotor synchronization generally benefits the perception of timing (Chemin et al., 2014; Nozaradan et al., 2016; Su & Pöppel, 2012) and would thus support a veridical percept of temporal structure in speech after a period of synchronized exposure (Manning & Schutz, 2013).
- Does an individual’s beat perception ability moderate their perceptual benefit from synchronized movement when encoding temporal structure of speech? We hypothesized that an enhanced beat perception skill goes hand in hand with an efficient encoding of the temporal structure and that strong beat perceivers do not necessarily require synchronized movement to guide their temporal processing (Su & Pöppel, 2012). In contrast, listeners with a low level of beat perception skill may not naturally and efficiently encode temporal structure without movement (Su & Pöppel, 2012). We therefore expected especially weak beat perceivers to show a perceptual benefit from synchronized movement

Addressing these questions can help to shed new light on the to-date unresolved issues outlined here – the p-center and the temporal regularization effect – by providing evidence for a range of phenomena potentially influencing malleability and fluidity of time perception in spoken language.

## Methods

### Participants

All participants of the study were recruited via Prolific Academic platform (www.prolific.co) (Peer et al., 2017; Douglas et al., 2023). Overall, 116 native British English speakers (58 female; mean age 41 years, range, 18–88) volunteered to take part in the study online, though only 107 of them completed all tasks. Twenty participants were excluded from further analyses because they did not follow the instructions of the study (i.e., they did not tap along with the stimuli in one of the experimental tasks), resulting in a complete dataset of responses containing the perception data of 87 participants in total (44 female; mean age 41.6 years, range, 18–88). Informed consent was obtained from all participants. Given a large age range in the sample, we ran an additional model to check for a potential effect of age and did not find sufficient evidence to suggest that age had any impact on participants responding “same” or “different” in the rhythm judgment task.

### Stimuli

Sixteen sentences were selected for the current experiment (see the Supplementary Materials). In contrast to previous research that studied either isolated words (Morton et al., 1976; Marcus, 1981; de Jong, 1992; Pompino-Marschall, 1989; Scott, 1998; Ryan, 2014; Harsin, 1997; Howell, 1988) or short phrases (Benadon, 2014; Darwin & Donovan, 1980; Donovan & Darwin, 1979; Lehiste, 1973), the present experiment used natural, complex sentences of English varying in length from minimally four to maximally 11 syllables, with two sentences selected for each number of syllables. The first author annotated the sentences manually using Praat (Boersma, 2001), identifying the onsets of each syllable and vowel. A Praat script was used to extract the time points of the annotated onsets along with the time points of the acoustic intensity maxima located within each syllable.

These timings were then used to create beat-based, drummed versions of each sentence. The beat was represented by a short (55-ms-long) sound of a drum, one per syllable of each sentence. Three drummed versions represented the temporal structure of each sentence, containing a series of drumbeats at the time points derived from (1) acoustic intensity maxima, (2) vowel onsets, or (3) syllable onsets. These veridical versions of the linguistic stimuli were complemented by (4) a regularized version of each sentence with a completely isochronous distribution of drumbeats matched to the duration of the sentence and its total number of syllables. Each test sentence was paired with all four drummed versions of its temporal structure, resulting in a total of 64 stimuli (16 x 4). Please note that the loudness of the two types of auditory stimuli was set to a comparable level, combining acoustic and perceptual tuning (first, a Praat script scaled both sounds to the same absolute peak; then, the two sounds were perceptually compared and the louder-perceived sound was step-by-step re-scaled until both sounded equally loud to the experimenters). One trial consisted of six repetitions of a test sentence separated by a 400-ms pause, followed by a longer pause of 1200 ms and finally a drummed version of the sentence (see Fig. 5).

**Fig. 1:**
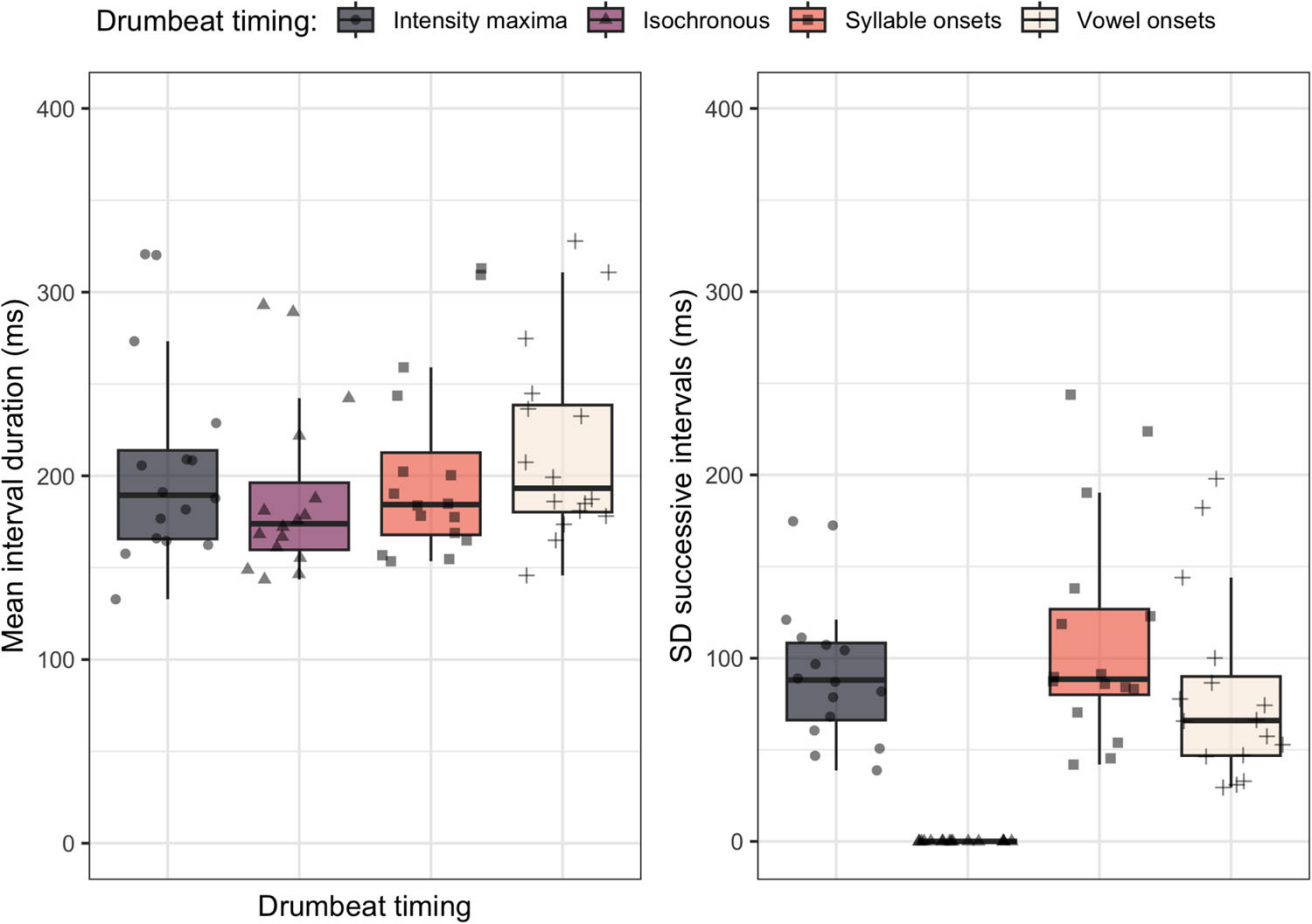
Mean interval duration and standard deviations of successive intervals between drumbeats (in ms) measured in the four drummed versions of test sentence

The temporal structure of the stimuli is compared in Fig. 1, showing mean interval durations and standard deviations of successive intervals as a measure of drumbeat variability across the different versions of the test sentences. As can be seen, mean interval duration (averaged over units, sentences and events) lied in the similar range across all stimuli, though intensity maxima ($\mu$ = 205.4 ms, $\sigma$ = 93.1 ms) and syllable onset ($\mu$ = 202.5 ms, $\sigma$ = 110.6 ms) intervals had a more similar mean duration than isochronous intervals that were a little shorter ($\mu$ = 189.5 ms, $\sigma$ = 0 ms) or vowel onset intervals that were a little longer ($\mu$ = 214.7 ms, $\sigma$ = 80.7 ms). We ran a Bayesian mixed-effect regression model to predict differences in mean interval duration by drumbeat timing, with the addition of sentence and sentence length as random intercepts. We found very strong evidence for a longer mean interval duration for intensity maxima (evidence ratio (ER) > 1999, posterior probability (PP) = 1.00), syllable onset (ER = 570.43, PP = 1.00) and vowel onset (ER > 1999, PP = 1.00) compared to the isochronous interval means. Very strong evidence also showed interval means of inter-vowel onsets to be larger than the means of intensity maxima intervals (ER = 55.34, PP = 0.98) and syllable onset intervals (ER = 499.00, PP = 1.00).

In contrast to the timing of the isochronous onsets, veridical intervals were more variable, though notably vowel onsets intervals displayed a slightly lower variability as compared to syllable onsets and intensity maxima onsets. We ran another Bayesian mixed-effect regression model on standard deviation of intervals measured for each of the four drumbeat timings, with sentence and sentence length as random intercepts. Very strong evidence showed higher variability for intensity maxima onset (ER > 1999, PP = 1.00), syllable onset (ER > 1999, PP = 1.00) and vowel onset (ER > 1999, PP = 1.00) compared to isochronous intervals. In addition, very strong evidence showed that the variability of syllable onset intervals was larger than the variability of vowel onset intervals (ER = 234.29, PP = 1.00).

**Fig. 2:**
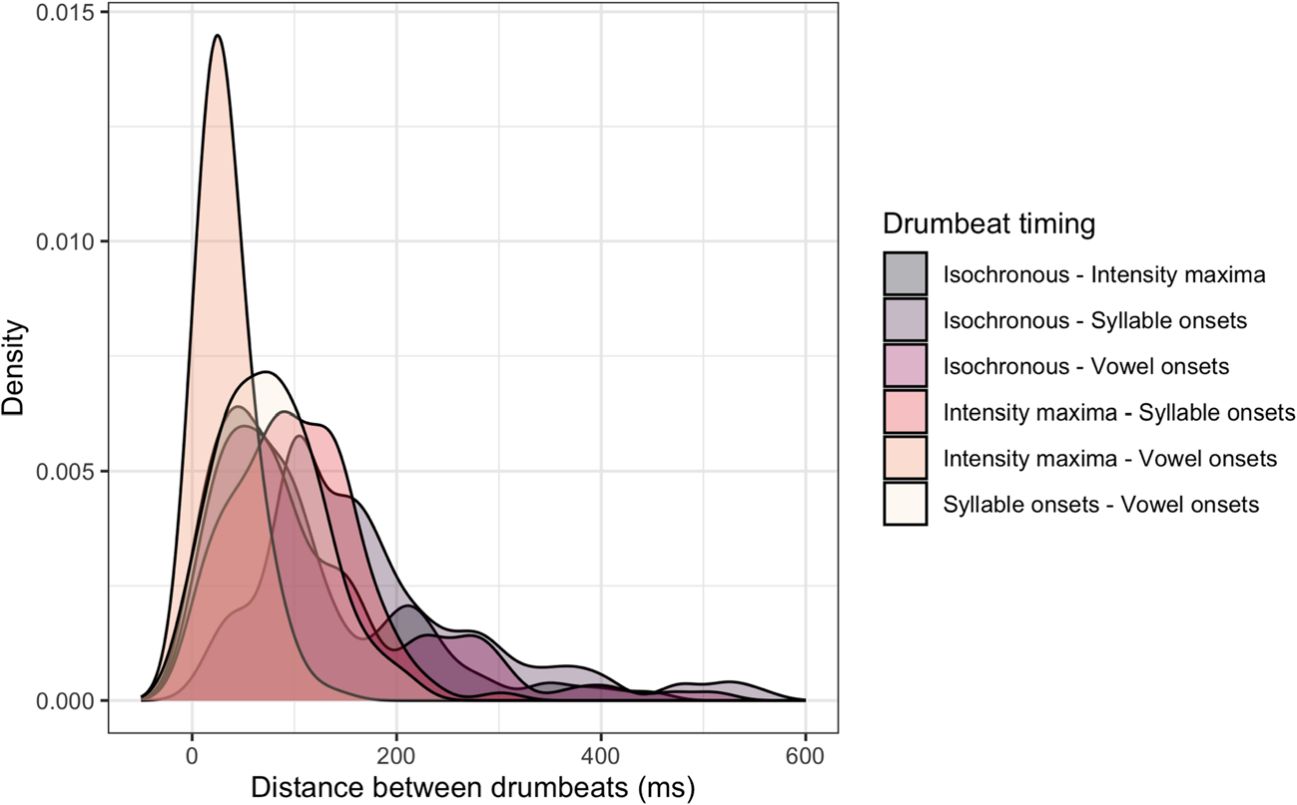
Temporal distance between drumbeats occurring in the same serial position across four experimental implementations of drumbeat timings (in ms)

Figure 2 compares temporal distances between drumbeats occurring in the same serial position across four experimental implementations of drumbeat timings. The distances are normalized with reference to the mean inter-onset interval duration of preceding intervals and shown as percentage of the corresponding inter-onset intervals (IOI). Among all comparisons, a very small number (47 or 7.5%) of all drumbeat pairs showed distances below the JND-threshold of 5% of the inter-onset interval duration (Friberg and Sundberg, 1995; Quené, 2007). The drumbeat pairs that did not meet the threshold comprised isochronous and vowel onsets (42 cases), isochronous and intensity maxima (1 case), intensity maxima and vowel onsets (4 cases). These comparisons indicate that most time series exemplified in the drumbeat stimuli of the present study meet the threshold criterion to be perceptually distinct, meaning that a potential lack of veridical perception in the present study cannot be explained by an increased perceptual similarity of the implemented drumbeat timings.

### Procedure

**Fig. 3:**
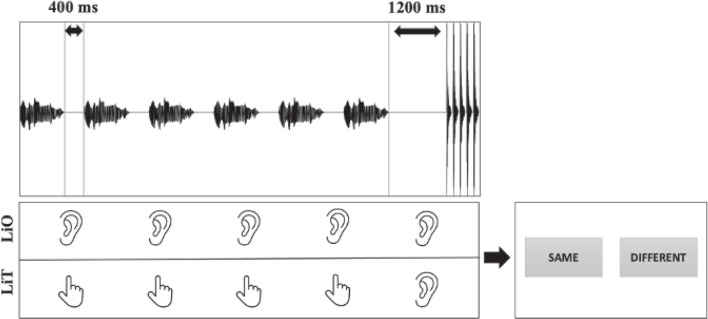
Summary of the experimental procedure consisting of a *Listen-Only* (LiO) vs. a *Listen-and-Tap* (LiT) exposure to speech stimuli (each repeated six times), followed by a longer silent pause and the drummed stimulus, concluded by a speeded same-different judgement. The example shows a five-syllable sentence and an isochronous sequence containing five drumbeats

The experiment consisted of two tasks, testing temporal perception in spoken and musical phrases. It started with the linguistic perception task in which we asked participants to compare the temporal structure of spoken sentences with their drummed versions and judge them as either “same” or “different” by clicking as fast as possible on one of the two response options appearing on the screen after a stimulus presentation. There were two types of exposure to the spoken sentences. During the *Listen-Only* exposure, participants’ task was to listen to six repetitions of each sentence quietly, compare the perceived sentence beat to one of the drummed sequences presented shortly after, and then respond “same” or “different” as soon as the two answer options were given on the screen. During the *Listen-and-Tap* exposure, participants had to synchronize with what they perceived to be the beat of each sentence during the six repetitions by tapping with the finger of their dominant hand on the touch pad of their device or by clicking on an external mouse attached to their device. They then compared the beat of the sentence to one of the drummed sequences played shortly after (without tapping) and had to judge the two versions as either “same” or “different” as soon as the two answer options were given on the screen. Note that the pause between sentence repetitions was shorter than the pause before the drummed version of the sentence was played (to prevent continued synchronization during the presentation of the drummed version of the sentence (Repp et al., 2008; Repp & Keller, 2004; Wing & Kristofferson, 1973)). A schematic representation of the experimental procedure is given in Fig. 3. The order of the two types of exposure was counterbalanced across participants.

The music task used in this experiment was the Computerised Adaptive Beat Alignment Test (CA-BAT) developed by Harrison and Müllensiefen (Harrison & Müllensiefen, 2018a, b). CA-BAT examines individual beat perception ability by asking listeners to spot temporal mismatches between a metronome beat and a musical extract. The test runs adaptively, starting with a misalignment that is easy to sport and successively tailoring the difficulty level to the individual performance of a participant during the test. Importantly for the purposes of the study, the adaptive test has a high level of granularity measuring individual sensitivity to temporal mismatches between the (overimposed) beat and the acoustic signal. This is a type of a beat alignment test (Dalla Bella et al., 2017; Fiveash et al., 2022; Iversen & Patel, 2008) that taps similar temporal processing mechanisms that are expected to be at play during speech perception task of the present study. Performance on such beat alignment tests is known to correlate across a range of sensorimotor and timekeeping abilities, including motor stability during unpaced tapping, accurate synchronization to an isochronous metronome sequence and accurate, stable reproduction in a synchronization-continuation task (i.e., continued tapping after the pacing metronome sequence had stopped (Dalla Bella et al., 2017)).

The individual BAT-index resulting from the CA-BAT test is a z-score normed with reference to the sample of the original study consisting of 197 participants (87 female) aged between 18 and 75 (mean age: 26 years, Harrison and Müllensiefen (2018a)). The score around 0 reflects an average beat perception ability, scores above 0 indicate an above-average ability, scores below 0 a below-average ability. In the present sample, 37 participants had a range of scores above 0, 40 participants had variable scores below 0, with three participants performing below 2 standard deviations of the group average. No participant was excluded based on their performance as long as they had completed all tasks following the task instructions (30 participants were excluded from the analyses because no taps were recorded during the *Listen-and-Tap* exposure of the linguistic task).

The experiment ran online, with the data from the linguistic task being collected on Gorilla (www.gorilla.sc) (Anwyl-Irvine et al., 2020, 2021) and the data from the musical task being collected on a local server (Harrison & Müllensiefen, 2018b). Participants were recruited and remunerated via Prolific Academic platform (www.prolific.co) (Peer et al., 2017; Douglas et al., 2023). They were instructed to use a tablet or a laptop computer while taking part and to play the sounds of the experiment through the built-in speakers of their devices (no wireless earphones or headphones were allowed). Once participants reached the end of the linguistic task on Gorilla, a new link opened on a separate page running the music task of CA-BAT (Harrison & Müllensiefen, 2018b). Given that the experiment was running online and unsupervised, it was set up to have a relatively short overall duration, intending to maintain participants’ full attention throughout the experiment. This was achieved by limiting the total number of linguistic trials per participant. Each participant completed 16 out of 64 trials (i.e., eight trials on each exposure type). They listened to each test sentence of the materials paired with one out of the four possible drumbeat versions of the sentence rhythm. An experimental session lasted no longer than 15–20 min. The protocol was approved by the Ethics Committee for the Linguistics Labs at the University of Konstanz (approval date: 04/02/2021) and the experiment was performed in accordance with relevant guidelines and regulations.

### Statistical analysis

To test the hypotheses of the present study, we used Bayesian multilevel regression models run in the statistical program R (R Core Team, 2021) with the brms package using Stan (Bükner, 2017, 2018; R Core Team, 2021). We tested the effects of three predictor variables (drumbeat timing, type of exposure in interaction with the individual BAT-ability) on the likelihood of the perception of temporal structure of speech being same as, or different from, the timing of drumbeats. That is, the dependent variable was coded as a binary response (same, 0 or different, 1). Three hypothesis-relevant models will be detailed below, though all modelling procedures were similar in that we started with a full model including all predictors of interest and with the random effects of Participant and Stimulus. We set a weakly informative prior with a Student’s t-distribution and three degrees of freedom, a mean of 0 and a scale of 1. We followed approximate leave-one-out (LOO) cross-validation to find the best-fit model for each of the hypotheses. In order to quantify the strength of evidence for each hypothesis, we used evidence ratios. These ratios are given by the posterior probability that the effect is in a hypothesized direction divided by the posterior probability that the effect is in the opposite direction (Smit et al., 2022). For the ease of interpretation, an evidence ratio of >19 is analogous to a *p* value of < 0.05. Such ratios are referred to as ‘strong evidence’ in a directional hypothesis testing using Bayesian regression (in contrast, the threshold for strong evidence is >39 for a bidirectional hypothesis, i.e., exploratory testing (Makowski et al., 2019)). The best-fit models are reported below.

## Results

### Individual tendency towards perceptual regularization

The first hypothesis of the study was tested by examining an individual tendency to regularize (i.e., to rate drumbeats with isochronous timing as being identical to the temporal structure of speech) as an effect of the individual BAT-ability under the *Listen-Only* exposure. To test this hypothesis, we conducted a Bayesian multilevel regression model on the perceptual ratings of isochronous drumbeats only with Response as the dependent variable, BAT ability as the predictor, Participant and Stimulus as the random effects. We found strong evidence (quantified by an evidence ratio (ER) > 19) for a positive effect of BAT ability in the *Listen-Only* condition (see Table 1). This means that the perception of temporal structure in the *Listen-Only* condition is less prone to regularization in those participants who have a higher BAT ability.

**Table 1:** Estimate = mean of the effect’s posterior distribution

Hypothesis	Estimate	[90% CI]	ER	PP
BAT ability > 0	0.85	[0.29, 1.51]	147.15	0.99

90% CI = 90% credibility intervals. ER = evidence ratio, or the odds that the effect is in the direction specified by the hypothesis. PP = the posterior probability

### Veridicality of the perception of temporal structure

The second model examined veridicality of the perception of temporal structure by testing the effect of the exposure type (*Listen-Only* vs. *Listen-and-Tap*) in interaction with drumbeat timing (isochronous vs. intensity maxima, vowel onset and syllable onset) on the binary response variable (same, 0 vs. different, 1). The model included Response as the dependent variable, an interaction between Drumbeat timing and Exposure as the predictor, Participant and Stimulus as the two random effects.

**Fig. 4:**
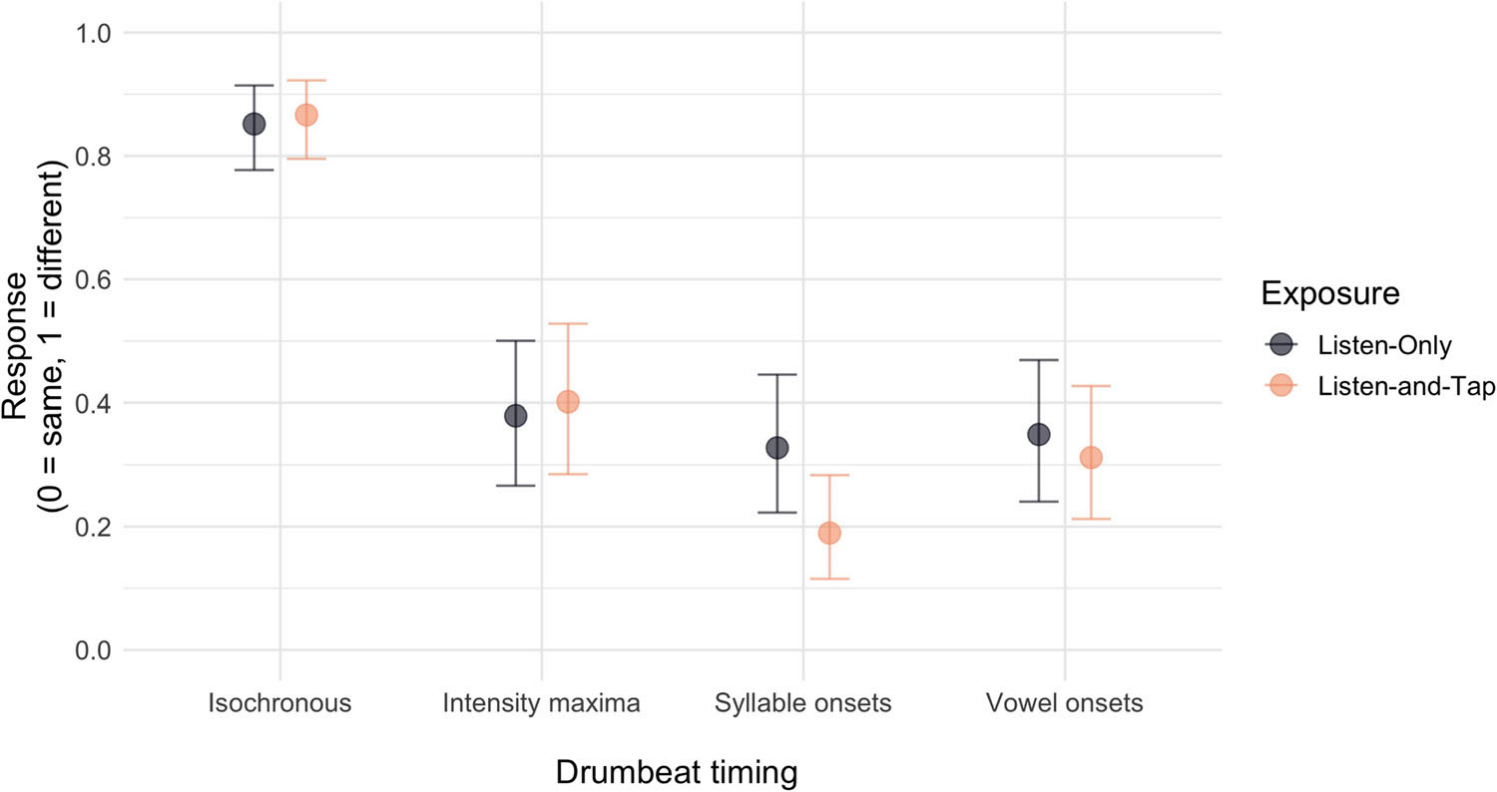
Conditional effects for the interaction of exposure type (*Listen-Only* vs. *Listen-and-Tap*) and drumbeat timing (isochronous, intensity maxima, syllable onset, vowel onset) on the veridical perception of temporal structure. The *errors bars* represent 95% credibility intervals around the predicted value of the response

Hypothesis testing shows that for the *Listen-Only* exposure, there is strong evidence that participants are more likely to rate speech and drumbeat as being different in the isochronous timing condition compared to the intensity maxima, syllable onset and vowel onset timing conditions. There is not sufficient evidence to support a difference in the hypothesized direction between the three veridical, non-isochronous conditions. For the *Listen-and-Tap* exposure, there is strong evidence for a difference in the hypothesized direction between all conditions, apart from a difference between the intensity maxima and the vowel onset condition. Comparing the four conditions between the two types of exposure, we find strong evidence that participants are more likely to rate the temporal structure of speech as the same as the drumbeats with the syllable onset timing after the *Listen-Only* exposure compared to after *Listen-and-Tap* exposure. There is not sufficient evidence to support a difference between the two types of exposure for any drumbeat timing. Results from the hypothesis testing are reported in Table 2 and the model output is visualized in Fig. 4.

**Table 2:** Estimate = mean of the effect’s posterior distribution

Exposure	Hypothesis	Estimate	[90% CI]	ER	PP
Listen-only	1. Isochronous < Intensity maxima	–2.28	[–2.74, –1.84]	>3999	1.00
	2. Isochronous < Syllable onset	–2.51	[–2.97, –2.05]	>3999	1.00
	3. Isochronous < Vowel onset	–2.41	[–2.88, –1.95]	>3999	1.00
	4. Intensity maxima > Vowel onset	0.13	[–0.28, 0.54]	2.35	0.70
	5. Vowel onset > Syllable onset	0.10	[–0.34, 0.53]	1.95	0.66
	6. Intensity maxima > Syllable onset	0.23	[–0.20, 0.65]	4.73	0.83
Listen-and-tap	1. Isochronous < Intensity maxima	–2.31	[-2.77, -1.85]	>3999	1.00
	2. Isochronous < Syllable onset	–3.38	[–3.88, –2.90]	>3999	1.00
	3. Isochronous < Vowel onset	–2.70	[–3.16, –2.26]	>3999	1.00
	4. Intensity maxima > Vowel onset	0.39	[–0.02, 0.82]	15.95	0.94
	5. Vowel onset > Syllable onset	0.68	[0.24, 1.14]	189.48	0.99
	6. Intensity maxima > Syllable onset	1.07	[0.63, 1.53]	>3999	1.00
Isochronous	1. Listen-and-Tap > Listen-Only	0.13	[–0.29, 0.56]	2.16	0.68
Intensity maxima	2. Listen-and-Tap > Listen-Only	0.15	[–0.78, 1.09]	1.53	0.60
Syllable onset	3. Listen-and-Tap > Listen-Only	1.00	[0.05, 1.96]	23.84	0.96
Vowel onset	4. Listen-and-Tap > Listen-Only	0.42	[–0.51, 1.34]	3.44	0.77

90% CI = 90% credibility intervals. ER = evidence ratio, or the odds that the effect is in the direction specified by the hypothesis. PP = the posterior probability

### Individual benefits from sensorimotor synchronization

The hypothesis that some individuals might benefit from synchronized movement more than others was examined in the final set of models, testing for the interaction of the individual BAT ability and exposure on regularized vs. veridical perception of temporal structure. For this, we ran a new set of models, one for each of the four drumbeat timing conditions. The structure of these models included a binary Response (same, 0 or different, 1) as the dependent variable, an interaction between BAT ability and Exposure as the predictors, Participant and Stimulus as the random effects.

Hypothesis testing for each of the drumbeat timings shows that among all veridical conditions, there is not sufficient evidence to document a difference between *Listen-Only* and *Listen-and-Tap* exposure on the perception of listeners with variable BAT abilities.

For isochronous timing, we find strong evidence for a perceptual change in response to the two exposure types in listeners with a low BAT ability (with a higher likelihood of choosing different under *Listen-and-Tap* than under *Listen-Only* exposure). This means that tapping while listening may be especially beneficial for listeners with a lower BAT ability, reducing the perceptual effect of regularization and promoting a more veridical percept.

**Fig. 5:**
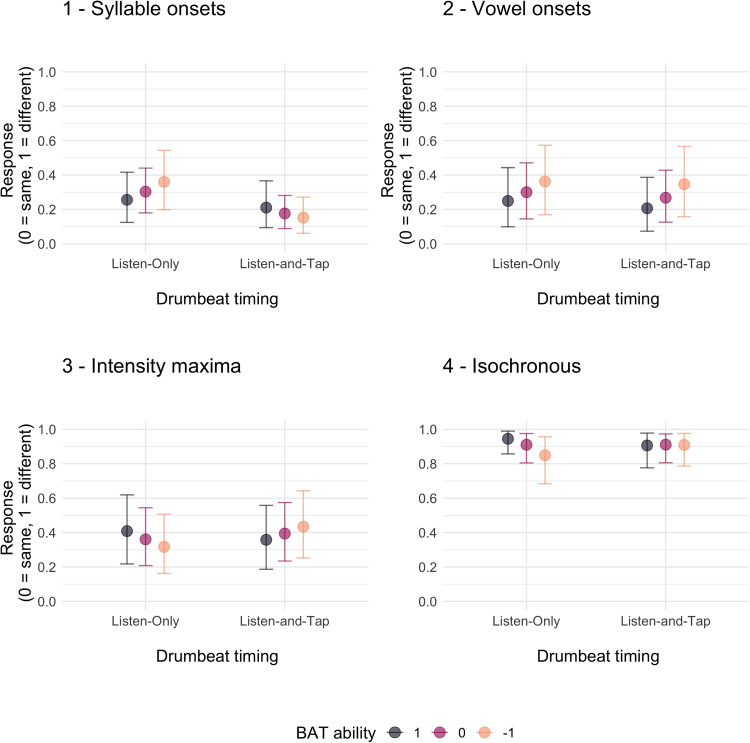
Conditional effects for the four drumbeat models displaying the interaction of exposure type (*Listen-Only* vs. *Listen-and-Tap*) and individual BAT ability. The errors bars represent 95% credibility intervals around the predicted value of the response

These results are summarized in Table 3. The output of the models is visualized in Fig. 5.

**Table 3:** Estimate = mean of the effect’s posterior distribution

Hypothesis	Estimate	[90% CI]	ER	PP
BAT ability (Listen-Only) < BAT ability (Listen-and-Tap)				
1. Syllable onset	–0.71	[–1.54, 0.11]	11.86	0.92
2. Vowel onset	–0.19	[–1.01, 0.65]	1.90	0.65
3. Intensity maxima	0.57	[–0.18, 1.36]	0.12	0.11
4. Isochronous	–1.21	[–2.22, –0.25]	59.61	0.98

90% CI = 90% credibility intervals. ER = evidence ratio, or the odds that the effect is in the direction specified by the hypothesis. PP = the posterior probability

## Discussion

The present study was conducted to examine the issue of temporal regularization that has been frequently documented for the perception of speech timing (Darwin & Donovan, 1980; Donovan & Darwin, 1979; Lehiste, 1973, 1977; Morton et al., 1976; Scott et al., 1985). When judging the duration of temporal intervals in spoken language, listeners tend to perceive them in a more regular fashion than their actual acoustic timings are, displaying a striking discrepancy between the acoustics and the perception of speech (Darwin & Donovan, 1980; Donovan & Darwin, 1979; Benadon, 2014; Scott et al., 1985; Rathcke et al., 2021). Such perceptual regularization is peculiar as it does not occur with non-speech sounds of a similar temporal structure (Darwin & Donovan, 1980; Lehiste, 1973; Scott et al., 1985; Benadon, 2014), thus making time perception in speech a fascinating, yet poorly understood, subject of study. To address it from a new perspective, we took inspiration in recent work on sensorimotor integration in improving auditory encoding and timekeeping (Chemin et al., 2014; Manning & Schutz, 2013; Nozaradan et al., 2016; Su & Pöppel, 2012) and examined the impact of synchronized movement and individual beat perception skills (Dalla Bella et al., 2017; Fiveash et al., 2022; Harrison & Müllensiefen, 2018a; Lagrois et al., 2019) on regularized vs. veridical perception of the temporal structure in speech. The results provide answers to the three research questions below.

*Does an individual beat perception ability play a role in perceptual regularization of speech intervals?* Using a rigorous measure of the individual beat perception ability - the Computerized Adaptive Beat Alignment Test (Harrison & Müllensiefen, 2018a, b), we provide strong evidence in support of the hypothesis that regularization relates to a low level of the rhythmic skill, supporting and extending previous research (Benadon, 2014). Strong beat perceivers, i.e., listeners who were better able to rate temporal mismatches between the (overimposed) beat and the signal in a range of music genres, were also less prone to perceptual regularization in speech. In contrast, weak beat perceivers showed a notable tendency toward perceptual regularization. The individual effect of the beat perception ability suggests that temporal processing in speech recruits a domain-general mechanism of internal timekeeping that links auditory processing of speech and music.

*Does sensorimotor synchronization enhance veridical perception of the temporal structure in speech?* The group-level results are in favor of our hypothesis. Following the *Listen-Only* exposure to speech, participants could merely distinguish between isochronous and non-isochronous drumbeat timings. This suggests they could reliably encode interval variability but not the overall temporal structure that includes interval duration, variability, and succession. After the synchronized exposure, group-level results support a more graded representation of all drumbeat timings, with veridical timings based on the acoustics (here, local intensity maxima) being rated as less reflective of the temporal structure of speech than veridical timings based on the linguistic events (syllable and vowel onsets). This suggests that body movement during listening enabled participants to establish a more graded percept of speech timing and promoted auditory encoding of the overall temporal structure and not just interval variability. In particular, participants rated speech to be more similar to those drumbeat timings that map onto the durations of inter-syllabic intervals, and there was very strong evidence for the role of the synchronized exposure in promoting this percept. Overall, the present evidence supports the conclusion that synchronized movement can promote perceptual encoding of the temporal structure of complex sounds (Chemin et al., 2014; Manning & Schutz, 2013; Phillips-Silver & Trainor, 2005, 2007, 2008; Su & Pöppel, 2012), and for the first time documents this facilitating effect in the perception of natural speech.

The reasons for the movement effect on perception are relatively poorly understood. The origins of the effect have sometimes been attributed to an interaction between the auditory and vestibular systems, which develops early in life (Phillips-Silver & Trainor, 2005, 2007, 2008; Trainor et al., 2009). Alternative suggestions (Manning & Schutz, 2013) raise the possibility that, instead of a vestibular effect, movement to the beat can improve listeners’ timing acuity and timekeeping, due to an increased attention to temporal regularities and an enhanced anticipation of the upcoming events (Large & Jones, 1999; McAuley & Kidd, 1998). A similar conclusion is reached in a study showing a perceptual benefit of synchronized movement for the processing of metrically regular speech (Falk & Dalla Bella, 2016). Other accounts suggest that moving in time with a sound engages the auditory-motor feedback loop which entrains to the regularities of sounds and thus supports the encoding of their temporal structure (Su & Pöppel, 2012). Without movement, the feedback loop has to rely on an internal motor entrainment which may pose great difficulties especially to musically untrained listeners (cf. Grahn & Rowe, 2009). Moreover, limited evidence demonstrates an amplified cortical and subcortical response to a sound’s temporal structure during movement, suggesting that neural entrainment might underpin the movement effect on perception (Nozaradan et al., 2016).

It has been widely discussed that sensorimotor synchronization capitalizes on the naturally occurring oscillatory brain frequencies that display moments of an enhanced excitability at specific points in time (Nobre & van Ede, 2018). Movement may moderate the alignment between the internal brain oscillations and the acoustic regularities, entraining neuronal excitations at the relevant timescales and thus enhancing the temporal encoding (Nozaradan et al., 2016). However, it is generally recognized that neuronal oscillations are periodic (Engel et al., 2001), and that body movement entrains to isochronous stimuli (Madison & Merker, 2002; Bolton, 1894). Accordingly, the movement effect on perception has been primarily studied with highly regular sounds in which isochrony is either present or implied. In contrast, our study demonstrates that the perceptual benefit of movement also exists with natural speech that inherently lacks isochrony (Dauer, 1983; Roach, 1982), thus challenging the isochrony-based accounts of the movement effect on perception. Given repetitions of spoken stimuli during exposure in our paradigm, regularities may have arisen and entrained brain oscillations on longer timescales. However, such timescales represent slower brain oscillation frequencies above and beyond the timescale of the beat that played an important role in explaining individual listener performance in the present study.

If neuronal entrainment drives the perceptual benefit of sensorimotor synchronization with natural speech, it is unlikely to be explained by a simple mechanism of a magnified oscillatory brain response at certain periodic frequencies. Instead, a complex interplay of amplitude and phase entrainment on multiple timescales that is indicative of the brain response to natural speech (Gross et al., 2014) may be enhanced through movement. The motor cortex exerts top-down influences that modulate the coupling phase of speech and low-frequency oscillations in the auditory cortex even without movement, during continuous speech perception (Park et al., 2015). Listening to a stream of syllables activates motor areas involved in speech production, which is consistent with the idea that the motor system is involved in processing of auditory spoken input (Wilson et al., 2004). Hence, movement synchronized with important acoustic landmarks of speech may facilitate the temporal sampling of the signal envelope irrespective of local timing fluctuations and regularities in the acoustic signal, potentially increasing the overall fidelity of auditory representations (Vanthornhout et al., 2018; Krause et al., 2010). Further studies comparing neural entrainment to speech signals after passive vs. synchronized listening could provide insights into the role of movement for speech perception in general.

In any case, understanding brain response to the temporal structure of natural speech is faced with the fundamental question which specific landmarks in the acoustic speech signals can brain oscillations lock on to. While it is commonly assumed that inter-syllabic intervals give rise to neural entrainment during continuous speech perception (Gross & Poeppel, 2019; Giraud & Poeppel, 2012; Peelle & Davis, 2012), empirical underpinnings of this assumption are yet to be fully addressed (Cummins, 2012; MacIntyre et al., 2022; Meyer et al., 2020). Our study shows that listeners cannot tell the difference between veridical temporal representations of sentences following a period of passive listening, despite the fact that acoustically, veridical timings of our stimuli differ from each other in substantial and noticeable ways (i.e., above and beyond JNDs reported in previous work (Friberg and Sundberg, 1995; Quené, 2007)). In contrast, a listening period accompanied by movement indeed makes listeners more likely to rate inter-syllabic intervals as being most representative of the temporal structure in speech. This finding is at odds with the results of sensorimotor synchronization experiments that show a stable motor entrainment to vowel onsets, with syllable onsets being the least likely anchor of synchronized movement (Rathcke et al., 2021). A similar discrepancy can be found in movement-based paradigms that ask listeners to tap out the temporal structure of speech after listening (Rathcke et al., 2021), indicating that synchronous vs. asynchronous tracking of inter-onset intervals may follow different principles.

*Do listeners with variable beat perception abilities benefit differently from synchronized movement?* We hypothesized that listeners with a high level of beat perception ability have a strong internal timekeeping mechanism (Tranchant & Peretz, 2020), resulting in a generally high fidelity of temporal representations. Thus, we did not expect strong beat perceivers to require synchronized movement in support of temporal encoding. In contrast, a low level of the beat perception skill is likely to be accompanied by a weak internal timekeeper and a relatively low fidelity of internally generated temporal representations. Thus, we expected weak beat perceivers to show a substantial perceptual benefit from synchronized movement. This hypothesis was partially borne out in the present study. We found strong evidence for a reduced perceptual regularization effect specifically in listeners with a low BAT ability. Listeners with a high BAT ability performed consistently and equally well after either type of exposure and generally showed little indication of a perceptual regularization effect. That is, the predicted effect was observed in the perception of isochronous drumbeat timings only, indicating diminished perceptual regularization upon movement in weak beat perceivers. The evidence was insufficient to document an individual benefit for the perception of veridical drumbeat timings.

Overall, present evidence corroborates previous findings obtained with simpler auditory prompts (Su & Pöppel, 2012), though instead of comparing groups of musicians and non-musicians as in previous research, we replaced a dichotomous view on individual variability and musical training by a graded approach to sampling listener abilities and examined the role of an isolated rhythmic skill - namely, beat perception - on the perceptual benefit of synchronized movement. Recent research emphasizes that individual musical abilities ought to be measured as a continuum instead of a dichotomy (Nayak et al., 2021; Tierney et al., 2021) as it eliminates the information about potentially meaningful individual differences (Cogo-Moreira & Lamont, 2018; MacCallum et al., 2002; Maxwell & Delaney, 1993; Royston et al., 2006). Even without having received formal musical training, individuals may have specific perceptual abilities (e.g., beat perception) commensurate with musicianship (Kragness et al., 2022; McKay, 2021; Swaminathan & Schellenberg, 2017, 2020; Wesseldijk et al., 2021). A graded approach to individual differences in the study of timing and time perception has a considerable theoretical and practical importance though most studies have so far focused on group-level effects (Matthews & Meck, 2014).

The present study implemented a laboratory task to study temporal perception in speech, by exposing listeners to repetitions of spoken sentences. The task bears little resemblance to the real life experience with continuous speech that requires listeners to encode time and timing on the fly, integrating incoming speech input into a coherent temporal representation of linguistic units. More naturalistic designs are required to test the scope of generalizability of the present findings to the real life settings. Such designs could examine the role of spontaneous co-speech gesturing and body back-channeling (Ambrazaitis & House, 2022; Habets et al., 2011; Cravotta et al., 2018) as well as individual beat perception ability on temporal encoding of continuous speech, with a similar set of predictions examined in the present study. The movement effect on temporal perception may influence prediction and comprehension of continuous speech and potentially play a role in language disorders (Goswami, 2011; Pagliarini et al., 2020).

Research into the perception of time and timing increasingly provides evidence for the lability of temporal judgments, supporting the idea that “time is a mental construction” (Pöppel, 1997 p. 56) and that “the brain is not like the measuring devices of classical physics” (Matthews and Meck, 2014 p. 429). The perception of temporal structure, an essential component of spoken language, testifies to this general malleability and fluidity of auditory perception (Benadon, 2014; Cooper et al., 1986; Fowler et al., 1988; Darwin & Donovan, 1980; de Jong, 1994; Donovan & Darwin, 1979; Fox & Lehiste, 1987; Marcus, 1981; Morton et al., 1976; Patel et al., 1999; Scott et al., 1985; Rathcke et al., 2021). The present study provides new evidence that perceptual judgements of time and timing in speech are affected by sensorimotor integration and individual beat perception ability.

### Supplementary information

The Supplementary Materials contain an overview of the sentences used in the study.

## Data Availability

The data sets generated and/or analysed during the current study are available from the corresponding author upon reasonable request.

## Code availability

The code generated to analyse the data are available from the corresponding author upon reasonable request.
